# Molecular Insights into the Biomedical Applications of *Plagiomnium affine* (Blandow ex Funck) T. Kop.: A Promising Source of Bioactive Metabolites

**DOI:** 10.3390/ijms26199341

**Published:** 2025-09-24

**Authors:** Julia Krupa, Andrzej Kaźmierczak, Izabela Kołodziejczyk

**Affiliations:** 1 Department of Geobotany and Plant Ecology, Faculty of Biology and Environmental Protection, University of Lodz, Banacha 12/16, 90-237 Lodz, Poland; julia.krupa@edu.uni.lodz.pl; 2 Department of Cytophysiology, Faculty of Biology and Environmental Protection, University of Lodz, Pomorska 141/143, 90-236 Lodz, Poland; andrzej.kazmierczak@biol.uni.lodz.pl

**Keywords:** bryophytes, bioactive metabolites, biomedical applications, cryoprotection flavonoids, *Plagiomnium affine*

## Abstract

*Plagiomnium affine* is a species of terrestrial moss that inhabits mainly coniferous forests but also occurs in areas with other characteristics. It is very adjustable, being a commercially available aquarium plant and popular among enthusiasts. Despite its wide distribution in various habitats, its physiological and biochemical adaptations, ecological roles, and responses to environmental changes remain only partially understood. In fact, it is not known what biomedical applications lie in this species, which is relatively easy to cultivate in vitro, and its role as an accumulator of elements such as manganese or cadmium is largely ignored. This article reviews the current state of research on *P. affine*, focusing on available published data that can help illuminate the biomedical application of the species, highlighting gaps in knowledge and identifying priorities for future research. For this purpose, all available literature, regardless of year of study, addressing the indicated topic was reviewed. The report presents *P. affine* as a uniquely adaptable moss species rich in bioactive compounds of scientific interest, highlighting its application importance in modern science.

## 1. Introduction

*Plagiomnium affine* (Blandow ex Funck) T. Kop. belongs to the *Mniaceae* family, commonly found in moist forests, wetlands, and shaded areas. As with other mosses, *P. affine* contributes significantly to ecological balance by absorbing and retaining moisture, which helps regulate local humidity and water availability, prevents soil desiccation, and benefits surrounding vegetation [1]. Moreover, mosses protect soil from erosion, particularly in forest understories and riverbanks. It also contributes to the accumulation of organic matter, thereby enriching soil fertility and habitat formation for microorganisms, insects, and small invertebrates. *P. affine* absorbs airborne pollutants and heavy metals, making it a valuable bioindicator for environmental monitoring, capturing atmospheric carbon dioxide [2,3]. Scientists have proven that *P. affine* contains numerous compounds that have properties for biomedical applications, such as antimicrobial, antifungal, anti-inflammatory, antioxidant, wound healing, and tissue regenerative engineering factors [4,5,6,7]. Extracts containing natural compounds exhibit antifreeze properties by preventing ice formation in the cells. This adaptive ability of *P. affine* also results in characteristic morphology of its leaves, with serrated edges and specific cell arrangements. The younger and the older leaves are less tolerant than intermediate ones, most likely due to a lower concentration of the cryoprotectants or specific cell structures that make them more susceptible to damage. However, much of its genetic, biochemical, and physiological adaptability remains unexplored, offering promising prospects for future research into both its environmental resilience and its biomedical applications. This moss species displays mechanisms enabling survival under extreme environmental conditions, particularly low temperatures [8,9,10,11].

The aim of this review is to summarize the current knowledge on *P. affine*, focusing on its morphology, regulation of local humidity, and bioactive compounds content, karyotype variability, and cold resistance strategies. Additionally, its adaptive properties and applications, including its use in aquaristics, are discussed. *P. affine* should also be considered in research due to its easy cultivation. This is possible in vitro from spores or by cloning under completely sterile or semi-sterile conditions on Knop’s nutrient solution (KN) or Murashige and Skoog’s nutrient medium (MS) [12]. This gives promising indications that moss could become a useful model plant. Despite its wide distribution, significant research gaps remain regarding its genetics, biochemistry, physiology, and environmental interactions, highlighting the need for further studies.

### 1.1. Methodology

To achieve the aim of this review, the literature was accessed and screened based on predefined inclusion and exclusion criteria (Table 1). In the initial screening, the publication year, title, and abstract were examined to identify relevant studies, while the full text of potentially eligible papers was subsequently assessed.

The systematic literature search was conducted following the PRISMA 2020 guidelines in four primary scientific databases: PubMed, Scopus, Web of Science, and Embase, and complemented by Google Scholar and ScienceDirect for additional references. The search covered the period from January to May 2025. A combination of controlled vocabulary (MeSH terms where applicable) and free-text keywords was used. An example full search string for PubMed was *P. affine*, (“Mosses” OR “Bryophytes”) AND (“Bioactive metabolites” OR “Medical Applications” OR “Flavonoids” OR “Cryoprotection” OR “DNA Content” OR “Chromosome Number” OR “Moss Life Cycle” OR “Taxonomy” OR “Rhizoids” OR “Polyploidy” OR “Genetics” OR “Karyology” OR “Ecology”).

**Figure 1:**
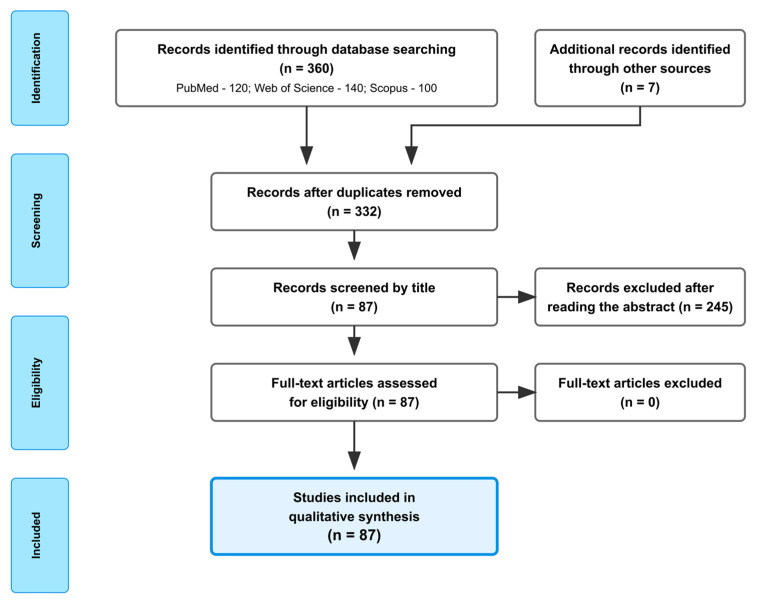
PRISMA flow diagram of study selection for systematic review on *Plagiomnium affine* research.

Equivalent search phrases were used across bibliographic databases (Scopus, Web of Science, and Embase) as well as publishing platforms (Springer, Elsevier, Wiley, Taylor & Francis, Oxford University Press, Cambridge University Press, Nature, Ibuk, and LEX). The systematic literature search was conducted following the PRISMA 2020 guidelines. A total of 360 records were initially identified through electronic database searching. After removing duplicates, 332 records were screened, and 87 full-text articles were included (Figure 1).

### 1.2. The Taxonomy of P. affine

Kingdom: *Plantae*; Phylum: *Bryophyta* (Mosses); Class: *Bryopsida*; Order: *Bryales*; Family: *Mniaceae*; Genus: *Plagiomnium* [1].

**Figure 2:**
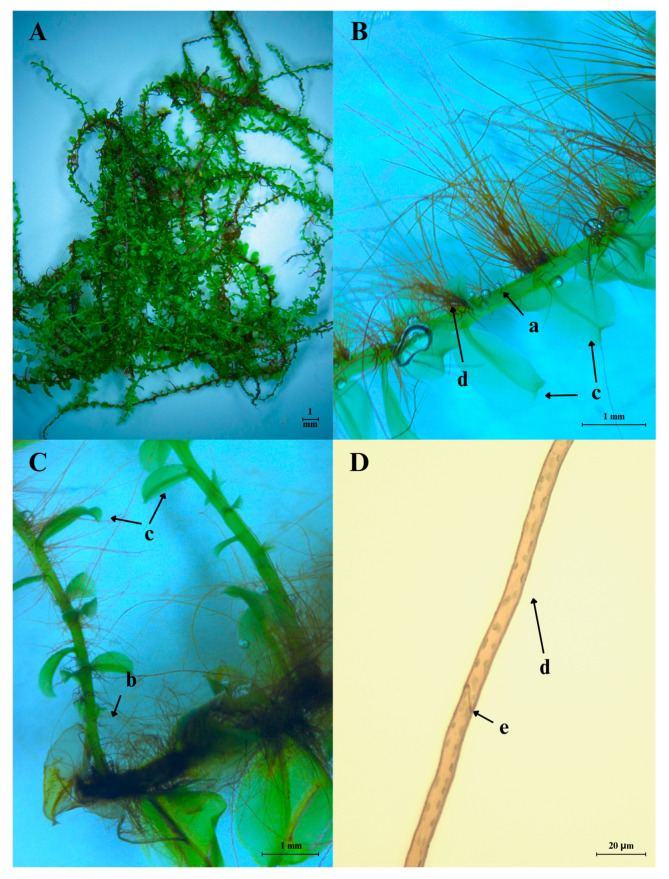
A clump of *P. affine* moss (**A**), unbranched (**B**) and branched (**C**) stems with spirally arranged (helically) leaves, and (**D**) rhizoids, as well as part of a rhizoid with a septum. a, unbranched stem; b, branched stem; c, leaves; d, rhizoids; e, septum.

*P. affine* (Figure 2) is a member of the *Mniaceae* family. These taxa divide into four different groups based on their sexual condition and morphology: there are (1) dioecious and monoecious counterparts distinguishable by morphological characteristics; (2) dioecious and monoicous taxa without distinct morphological differences; (3) predominantly monoicous species with diploid chromosome numbers, lacking known dioecious counterparts; and (4) the taxa consisting exclusively of dioecious plants. The *Mniaceae* includes nine genera and 74 species [1,13,14,15,16,17,18,19,20,21].

Genetically, certain monoecious species are believed to be autodiploids, whereas others are allopolyploids resulting from hybridization between distinct species [1].

Most monoecious species in the *Mniaceae* family have broad geographic distributions, although some are endemic to isolated regions. In contrast, dioecious species exhibit varying distribution patterns: some have a widespread Holarctic range, while others are confined to forested areas of temperate and southern North America, Europe, Southeast Asia, or subtropical Asia [1].

The species *P. affine* was previously classified under the genus *Mnium*, but molecular and morphological studies led to its reclassification under *Plagiomnium,* and it is often confused with *Plagiomnium elatum* and *Plagiomnium ellipticum* [22].

*P. affine* (Blandow ex Funck) T. Kop. is a species of moss that occurs relatively widely in moist but not flooded boreal forests on the European, Asian, and North American continents, although it can also be found in meadows and on lawns. It prefers alkaline to slightly acidic soils [23,24]. The wide occurrence of the species in various conditions, although with preferences, arouses our curiosity about the features that are responsible for their ability to adapt, but have not yet been explained.

One of the main challenges with the initial classification of *Plagiomnium* species was their pronounced morphological similarity. As shown by Lindberg in 1868 [25], *P. affine* and *P. ciliare* were previously considered to be the same species. Mamczarz, 1974 [26], presents detailed structural differentiation between *P. affine* and *P. elatum*. A notable feature of *P. affine* is the generally higher number of cells in the serrated leaf margin as well as their length. Despite these differences, the midrib and the central part of the lamina exhibit considerable similarities [26].

*P. affine* has been studied in aspects of taxonomy and ecology. However, genetics and physiological adaptations, environmental interactions, and stress responses need to be completed (author comments).

### 1.3. Morphology and Reproductive Biology of P. affine

Vegetative shoots (gametophytes) of *P. affine* grow prostrate and reach approximately 2 cm in length and have dense foliage, often forming extensive colonies. When present, the fertile shoots are unisexual. After fertilization, an erect, diploid sporophyte emerges from the female shoot. Through meiosis, it produces haploid spores that can be dispersed by the wind over long distances. In Northern Europe, sporophytes that produce capsules are uncommon [27]. Each shoot produces one to five sporophytes, which are supported by bright red bristles. The capsules, yellowish to yellowish brown, are ovoid or ellipsoidal and have no distinct neck. Inside the capsules are spores 15 to 24 μm in size, closed by a short, conical cap [28,29]. The species is dioecious [24] with two types of stems: sterile and fertile. Sterile stems have curved, rooting ends and complex but sparser leaves. Fertile stems are distinguished by larger leaves, usually concentrated at the top of the structure. Sterile stems can reach up to 10 cm, while fertile ones usually reach approximately 6 cm [10,11,28,29].

The leaves have a short, pointed apex and are broadly elliptic; however, they also occur as obovate or lanceolate. Regardless of leaf shape, depending on the habitat, the margins can be flat or spinosely serrate, featuring sharp teeth arranged at various angles, each consisting of up to four or more cells. The leaf base extends down the stem, a common trait in this genus [30]. Apical leaves are usually mucronate. Upper leaves are oblong to tongue-shaped, while lower leaves are rather broadly elliptic to rounded. Regardless of the position, the leaf edges narrow and converge toward the stem base. Leaf tips can be blunt or semi-blunt, with a vein that extends either to the apex or to the ends [28,29,30].

The leaf cells are isodiametric and pitted [31]. It should be noted that the width of the central leaf cells ranges from 30 to 45 μm, while their length is almost twice as long. These cells form porous, hexagonal rows that are aligned toward the leaf midrib [28,29].

The presence of well-defined stereid cells in the midrib is a distinguishing feature of *P. affine* and other *Plagiomnium* species, like *Plagiomnium guizhouense*, which is remarkably like *P. affine* [30].

When dried, the leaves of *P. affine* roll up strongly, while in a humid environment, they remain flat and spread out [31]. *P. affine* can function in both terrestrial and aquatic environments due to its tolerance for changing environmental conditions and due to the absence of a typical root system, having only rhizoids. Particularly crucial for moss adaptation is the ability to reproduce both on land and in water, and spores are dispersed by wind and water. The leaves of *P. affine* are hygroscopic, do not have a typical cuticle, and are highly dependent on the environmental humidity, so most of them do not need regulated gas exchange, like vascular plants. Therefore, many species of mosses, including *P. affine*, have not developed stomata [32,33,34].

### 1.4. Karyology of P. affine

Analyses of five populations of *P. affine* in Poland indicated that the observed chromosome numbers (n) are 6, 7, 12, and 18 [1,35]. This variation likely results from polyploidization events, as well as structural chromosome changes, including both metacentric and submetacentric forms and occasional mutations [1,35].

Polyploidy in the studied populations, as well as their genetic differentiation, was confirmed by examining allozyme variation across 23 loci, which provided clear evidence of variation at the genetic level [27].

In contrast, European populations generally exhibit a uniform chromosome number of n = 6 [36].

Although the nuclear DNA (C) content of *P. affine* has not yet been determined, other species within the *Mniaceae* family display values ranging from 0.5 to 2.5 pg [1,21]. However, no data are currently available to determine the nuclear DNA (C) content specifically for *P. affine*.

## 2. Physiological and Chemical Properties of *P. affine*

*P. affine* has attracted scientific interest due to its rich profile of secondary metabolites [37] and unique biochemical adaptations that support survival under variable environmental conditions [3]. Understanding the physiological traits and chemical composition of this moss is essential for evaluating its biomedical applications and adaptive strategies at the molecular level.

### 2.1. P. affine as a Bioindicator

Studies of 26 species strongly suggested that bryophytes in the East Tatra Mountains accumulate certain heavy metals. Studies indicated that in *P. affine*, the concentrations of cadmium and manganese increased [3]. Additional studies of mosses collected in the West Carpathians in the autumn of 2012 showed that sulfur, zinc, chromium, manganese, molybdenum, calcium, and copper are primarily accumulated in the capsule. Potassium and strontium accumulated in the sporophyte, while lead was found mainly in the gametophyte, zinc in the gametophyte stem, and iron in the older-than-one-year segments [2]. These results indicate that the mosses can be used as bioindicators for biomonitoring heavy metal pollution. Studies carried out between 1990 and 2005 on European mosses indicated that the lowest metal concentrations occurred in mosses from Scandinavia, the Baltic States, and northern parts of the UK. In Belgium and South-Eastern Europe, the highest concentrations of arsenic, cadmium, iron, lead, and vanadium decreased by approximately 52–72%, whereas copper, nickel, and zinc decreased by approximately 20–30%. A reduction in mercury of approximately 12% and in chromium of about 2% has been observed since 1995 [38].

### 2.2. Role of P. affine in Regulation of Local Humidity

Mosses regulate local humidity and prevent both soil and their desiccation by absorbing water directly through large, empty (dead) hyaline cells in leaves and stems, which act like sponges, forming a water reservoir. The stored water is gradually released to surrounding tissues, helping to maintain local humidity during dry periods and winter [39]. For *P. affine*, there is a lack of detailed studies confirming the presence and role of hyaline cells in water storage. However, other structures, such as papillae on leaf surfaces or specific features of cell walls, pores, and capillaries, which may also play a significant role in water retention, are observed in cells of leaves [40]. The water retention may also be regulated by the tiny filamentous or plate-like paraphilia and lamellae, outgrowths on the stem and leaf surfaces [41].

Mosses, including *P. affine*, also create a dense mat that traps water within its structure. By forming a barrier, moss slows down evaporation and prevents rapid moisture loss from the soil caused by sunlight and wind exposure, thereby reducing soil temperature fluctuations, keeping it cooler during hot periods, and insulating it during colder periods. In ecosystems prone to seasonal droughts, the moss acts as a buffer, improving soil resilience against prolonged dry periods. This function is particularly important in meadows, forests, and urban environments where rapid desiccation can negatively impact biodiversity [42].

### 2.3. Cryoprotective Properties and Frozen Sensitivity of P. affine

Another important property of *P. affine* is its cryoprotective capacity. Research indicates that cryoprotection is achieved through several mechanisms involving the uptake, especially of sucrose, glucose, and DMSO (dimethylsulfoxide). In contrast, glycerol, polyethylene glycol, and proline reduce their effectiveness as cryoprotection due to plasmolysis induction and, in the case of proline, low permeability through the plasma membrane [11].

*P. affine* has also been studied in terms of frost sensitivity regarding the age of leaves [10]. The leaves were frozen, and following the thawing process, parameters associated with the quality of photosynthesis were estimated using fluorescence methods, and the kinetics of chlorophyll were assessed. As previously reported, results revealed that the youngest and oldest leaves have lower freezing tolerance in comparison with mature or intermediate leaves. A notable conclusion was that the inactivation of the photosynthetic process is equivalent to that observed in higher plants. Freezing gradually inactivates the electron transportation in photosystem II (PSII) by inhibiting photoreaction. In *P. affine*, the transfer of excitation energy between pigment antennas and PSII cores was disrupted. As anticipated, after thawing, the leaves showed necrotic changes. However, the cells maintain the ability to undergo plasmolysis and deplasmolysis [10].

Overall, the data indicate that mature leaves show significantly better frost tolerance than younger leaves. After freezing, a comparison of oxygen uptake between mitochondrial respiration and photosynthesis indicates that the latter is considerably more sensitive to freezing-induced damage. In the event of lethal exposure to low temperatures, the tonoplast and the cell membrane sustain irreversible damage, causing a loss of cell turgor [10], thus decreasing the probability of plant survival.

Analysis of sugars from various stem parts and leaves revealed that differences in individual development and frost resistance cannot be attributed to the differences in glucose, sucrose, and fructose levels. Consequently, the research hypothesis suggesting such a dependence was disproved. Especially since mature leaves that maintained the highest level of photosynthesis did not show an increased sugar content in comparison with other leaf types. Freezing and thawing had minimal impact on mature leaves. However, it reduced the net photosynthetic rate in older leaves, while it severely damaged the photosynthetic apparatus in young leaves, confirming them to be the most vulnerable. The crystallization of water into ice has also been observed in those leaves. It would be more suitable to propose that the efficiency of photosynthesis causes sugar abundance and reduction impacts the usage of reserve sugar during starvation conditions, rather than relying on the previously mentioned hypothesis [10,11].

Nevertheless, the specific conditions under which the species can survive even freezing winters remain unclear [10], and this question has not yet been resolved. A candidate chemical marker identified in *P. affine* is n-octadecane, a compound widely used in industry, for example, in waxes, lubricants, and phase-change materials for heat storage [8,9].

### 2.4. P. affine Is a Source of Bioactive Metabolites

The presence of specific compounds in plants can provide insights into the types of stress they encounter. Certain groups of compounds may be regarded as beneficial from a human perspective, with applications in agriculture, pharmacology, medicine, and other fields. Higher plants have been extensively studied; mosses are now receiving increasing attention for their biomedical applications [43,44,45,46,47,48,49,50,51,52,53,54,55,56,57,58,59,60,61,62,63,64,65,66,67,68,69,70,71].

Important work in this context was initiated by Suire et al., 2000 [37]. The research team indicated specific chemical components of 13 different moss species. During the experiment, three categories of compounds were identified: primary and trace components and metabolites falling between both of those mentioned groups. The main component identified in *P. affine* was phytol, while the remaining metabolites were only present in trace amounts, including α-tocopherol, squalene, hop-22(29)-ene, palmitic acid (C16), methyl 2,4-dihydroxy-3,6-dimethyl benzoate, aromatic compounds, and flavone O-glycosides [37].

A separate study focused primarily on the flavonoids present in *P. affine* [44]. It was found that gametophytes contain five distinct flavonoid glycosides, including isoorientin and other O-glycosides, since during acid hydrolysis, both glucose and isoorientin were detected. In the case of the remaining flavonoids, nearly identical results were observed during a chromatographic analysis. The presence of isoorientin 3′-O-sophoroside was also confirmed, and further analyses characterized the flavonoid as isoorientin 3′-O-neohesperidoside.

Some studies [37,43,44], focusing on the biomedical applications of mosses to a certain extent, have also included *P. affine*. The number of publications is limited, but they do provide at least minimal information regarding the bioactive metabolites present in the mentioned species (Table 2).

In *Plagiomnium* sp., metabolites such as biflavones, dihydroflavonols, and C- and O-glycosides have been identified. Antibacterial, antifungal, and phytotoxic effects and protective properties against UV radiation have also been detected [7].

For instance, Altuner et al. 2011 [6] indicated that *P. affine* exhibits a static type of activity containing *Salmonella enterica* serotype *Typhimurium* SL1344 during tests, although this may be attributed to the insufficient concentration of the substance in the stock solution. The industrial use of mosses in fields related to human health also encompasses studies that have granted the possibility of identifying triterpenoidal saponins in *Plagiomnium* sp., which exhibit a beneficial effect on infections. Additionally, this compound is applied to reduce swelling and even serve as a base material for plaster used in fracture treatment [5].

In *Plagiomnium* sp., bicyclohumulenone, menthanemonoterpenoids, plagiochiline A, plagiochilide, plagiochilal B, ricardins A and B, sacullatal, and triterpenoidal saponins that exhibit antioxidant properties have been identified (Table 2) [55,56]. The presence of the latter compound has been confirmed by the findings of Mishra et al., 2014 [5] (Table 2).

Mosses most likely exhibit high bioactive metabolite activities, as they are generally unharmed by microorganisms and avoided by insects and pests. From *P. affine* extracts, flavonoids such as apigenin, isoorientin, isoorientin 3′-O-neohesperidoside, isoorientin 3′-O-sophorosiole, and vitexin were isolated. In traditional Chinese medicine, the mentioned mosses are known to effectively treat bacterial infections. Bioactive metabolites identified in *Plagiomnium* sp. and their importance for humans are demonstrated in Table 2.

In studies of bryophytes investigating the antibacterial and antiproliferative activities of moss metabolites, specimens were collected in the Northern Medium Mountains of Hungary in 2014, and voucher specimens were deposited at the University of Szeged. The air-dried, powdered plant material was extracted and fractionated into four types of extracts (A–D). First, the material was macerated with chloroform (Extract A) at a 1:10 *w*/*v* ratio for 24 h at room temperature. After filtration and solvent removal, the residue was sequentially extracted with methanol to obtain Extract B, with n-hexane to produce Extract C, and finally with water or aqueous alcohol to yield Extract D. Each extract was filtered and evaporated under reduced pressure to obtain the final dry extract.

Examining the antimicrobial properties allowed the scientists to determine that extracts derived from *P. affine* (prepared using CHCl_3_) exhibit notable activity against *B. subtilis* and *S. pneumoniae* (respectively, inhibition zones of 8.0 mm and 8.5 mm). The antimicrobial activity of bryophyte extracts, tested by the disc-diffusion method against 11 standard strains, was generally weak and sporadic. Out of 42 species, only 19 samples from 15 taxa showed moderate antibacterial effects, with no activity against *Pseudomonas aeruginosa*, *Escherichia coli*, or *Klebsiella pneumoniae*. The most susceptible strains were methicillin-resistant *Staphylococcus aureus* (MRSA, ATCC 43300) and *Staphylococcus aureus* (ATCC 29213). Antibacterial effects were observed mainly in the less polar n-hexane and chloroform fractions, while aqueous and methanolic residues were inactive. *Plagiomnium cuspidatum* showed the broadest activity, being effective against eight strains, and thus represents a promising candidate for further investigation. A correlation between antiproliferative and antimicrobial activities was also noted.

The anti-proliferation activity of moss extracts screened in vitro was demonstrated using HeLa, A2780, and T47D cell lines with the 3-(4,5-dimethylthiazol-2-yl)-2,5-diphenyltetrazolium bromide (MTT) assay. They indicate a significant value of this plant for biomedical applications [71]. The antiproliferative activity of these extracts was assessed in vitro using the MTT assay on human gynecological cancer cell lines: A2780 (ovarian), HeLa (cervical), and T47D (breast). Cells were treated with the extracts (10 and 30 µg mL^−1^) for 72 h, and viability was measured spectrophotometrically. Stock solutions were prepared in 0.3% DMSO, which had no significant effect on cell proliferation, confirming that the observed activity was due to the extracts themselves. Cisplatin served as a positive control, with IC_50_ values of 1.30 µM (A2780), 12.43 µM (HeLa), and 9.78 µM (T47D).

Table 3 shows the antiproliferative effects of the *P. affine* extracts on different cancer cell lines.

## 3. Conclusions

Given the time frame in which the referenced studies were conducted and published, it is evident that this topic has not been widely explored by contemporary researchers. Despite *P. affine* being a common moss, frequently found in nature and used as an aquarium plant, including biomedical and biochemical applications, it remains largely unexplored. Crucial factors underlying its phenotypic plasticity remain uncharacterized, and its chemical composition and the molecular mechanisms responsible for adaptation are insufficiently researched. This lack of information can lead to speculative or ambiguous discussions regarding the species.

The limited scientific literature collected in this work aims to provide a foundation for future research, particularly for scientists interested in the biomedical and beneficial properties of mosses. Among them, *P. affine* could be used as an alternative to other species that are currently attracting scientific interest.

Furthermore, due to the significant time difference between the original studies and the present, it remains uncertain whether their findings should be repeated, given environmental variability. In the context of global climate change, some of the previously established information may no longer be applicable. To fully understand *P. affine*, extensive research under controlled conditions is required, particularly to assess its role as a model plant and its broader applications, including as a resource for the acquisition of desired bioactive metabolites.

One of the most important metabolites in this context seems to be phytol, identified in *P. affine*. The bioactive metabolite has been widely studied in higher plants and other mosses [82,83,84,85] and exhibits a range of biological activities, making it a candidate for pharmaceutical and cosmetic applications: It has anti-inflammatory properties, inhibiting inflammatory mediators (e.g., TNF-α, IL-6), as confirmed in cell and animal models. It has anticancer properties, inducing apoptosis in cancer cells (e.g., breast and prostate cancer). It acts, among other things, through the mitochondrial pathway, regulating Bcl-2 and caspase activities. It has antioxidant properties, reducing oxidative stress and protecting against free radical damage. It has antibacterial and antifungal properties, inhibiting the growth of certain strains of bacteria (*Staphylococcus aureus*) and fungi (*Candida* spp.). It also exhibits a sedative (neuroprotective) effect; in mice studies, phytol exhibited hypnotic and sedative effects through interaction with the GABA receptor. Phytol is also a precursor of vitamin E (α-tocopherol) and vitamin K; it can be used for the semi-synthesis of drugs or supplements [56,82,83,84,85].

The situation is similar for N-octadecane, although it is also not a unique bioactive metabolite for *P. affine*, which is an interesting cryoprotectant and may have applications in the cosmetics and pharmaceutical industries. N-alkanes serve as formulation ingredients, e.g., as stabilizers, nonionic emollients, and carrier ingredients in cosmetic preparations. A producer of natural n-octadecane may be an alternative to obtaining synthetic raw materials [86,87,88].

So why might the production of these metabolites by *P. affine* be important? Bryophytes are a sustainable source, as they are simple organisms that do not require intensive cultivation. They can grow in sterile media without pesticides or fertilizers. Production in vitro or in bioreactors is possible, demonstrating the high plasticity of *P. affine* as a species common in the wild and in aquariums. This is important for bioengineering and plant pharmaceuticals, as it allows for the selection of material for cultivation under controlled conditions to produce phytol and other secondary metabolites.

*P. affine* can be used as an alternative source of this compound, especially if isolation from higher plants is costly or environmentally burdensome. The species is suitable for research on natural medicinal products, particularly in the context of bryophyte bioprospecting. Therefore, it deserves further investigation as a potential platform for biofactory applications to produce bioactive metabolites.

## Figures and Tables

**Table 1:** The exclusion and inclusion criteria in the literature search.

Criteria	Description
Exclusion	Editorials, letters, books, encyclopedias, non-English publications, duplicate publications, conference abstracts without full data
Inclusion	No restriction on study location; studies on bryophytes (mosses) addressing one or more of the following: bioactive metabolites, biomedical applications, occurrence of flavonoids or other secondary metabolites, cryoprotective properties, DNA content, chromosome number, life cycle, taxonomy, rhizoids, polyploidy, genetics, karyology, ecological functions

**Table 2:** Bioactive metabolites identified in *Plagiomnium affine* and other mosses of the *Plagiomnium* genus, along with their chemical structure and medicinal, pharmaceutical, and cosmetic properties. Superscript numbers indicate the respective items listed in the Supplementary Materials (Boxes S1–S9).

Metabolite	Molecular Monoisotopic Masses in Daltons (Da) **	Properties/Functions Important to Humans	Bibliography
**Acyclic hydrogenated diterpene alcohol**			
Phytol *** ** 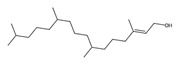 **	≈296.3079	It is suggested to have antinociceptive, antioxidant ^1^, anti-inflammatory ^2^, and anticarcinogenic ^9^ properties, as well as an anticonvulsant and anxiolytic-like agent.	[37,51]
**Aliphatic hydrocarbons**			
n-Octadecane ** 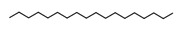 **	≈254.2974	A component of cuticular waxes in plants and secretions of some microorganisms; used in industrial applications as a compound of lubricants, waxes, and phase change materials for heat storage.	[37]
n-Heptacosane 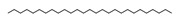	≈380.4382	Natural saturated hydrocarbon, lipid derivative, anti-inflammatory ^2^ properties, and antibacterial ^8^ activities; in the cuticle of leaves, fruits, and seeds of plants, protects against water loss and acts as a barrier to pathogens; they are also present in animals, especially insects. Applied in skincare and haircare cosmetics.	[37,72]
**flavonoids**			
Isoorientin *** 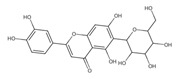	≈448.1006	C-glucosyl flavone, antioxidant ^1^, anti-inflammatory ^2^, antidiabetic ^3^, and anti-obesity ^4^ agent.	[44,45]
Isoorientin 3′-O-sophoroside *** 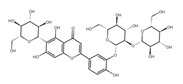	≈790.2166	Antioxidant ^1^ and anti-inflammatory ^2^ can be applied in nutraceutical formulations and cosmetic products. For the mechanism of action and biomedical applications, further research is required.	[44,46]
Isoorientin 3′-O-neohesperidoside *** 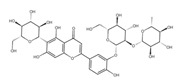	≈756.2113	Antioxidant ^1^ modulator, anti-inflammatory ^2^, and cytoprotective agent; may be considered as a dietary supplement or an ingredient in food and cosmetics.	[44,47]
**phenolic compounds**			
Methyl 2,4-dihydroxy-3,6-dimethylbenzoate (atraric acid) * 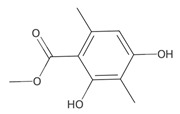	196.1999	Anti-androgenic agent, components in perfumery, cosmetics, detergents, fabric softeners, candles, and incense; concentrated may cause mild skin irritation.	[37,48,49]
**sesquiterpenes/sesquiterpenoids**			
*β*-Bisabolene (tentatively identified) 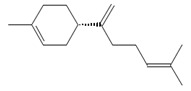	≈204.3511	Naturally present in plants, it can be applied as an anti-inflammatory ^2^, antibacterial ^8^, and anticancer ^9^ (for breast cancer) factor, especially against *Staphylococcus aureus*, in the pharmaceutical and cosmetic industries.	[37,57]
*δ*-Cuparenol 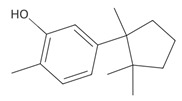	≈219.1747	Primarily isolated from the liverwort *Bazzania pompeana*, an agent with biomedical applications, especially as an anti-inflammatory ^2^ and antibacterial ^8^ activities, can be used for skin protection.	[37,58]
ent-*β*-cedrene * 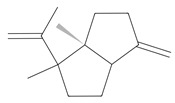 *	≈204.1878	Anti-inflammatory ^2^, antiseptic ^8^, and antispasmodic essential oil evoking sedative, diuretic, insecticidal, tonic, and astringent effects.	[37,59]
*α*-Cedrene 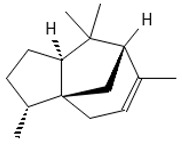	≈204.1878	Natural essential oils (e.g., cedar, juniper, cypress) with anti-inflammatory ^2^ and analgesic properties with arthritis properties are also applied as antiseptic ^8^ agent, suggested as anticancer ^9^ metabolites (e.g., anti-mouth, liver, and lung cancer), and as fragrances, and they are used in cosmetics and as the dainty sweet taste in the food industry.	[37,60]
*α*-Acoradiene * 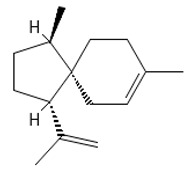 *	≈204.1878	Natural essential oil with aromatic properties and antioxidant ^1^ activities; it plays a vital role in protective and regenerative mechanisms as part of inflammation defense, immune processes, and antibacterial ^8^ and antiviral properties; it is applied in cosmetics (for skin and hair) and pharmaceutical industries.	[37,61]
**diterpenoids**			
Dolabella-7,8-dien-18-ol ** 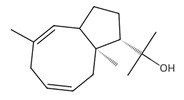 **	≈290.2609	Essential oil of, e.g., *Pseudocorythion acutum* may present antioxidative ^1^ as well as anti-inflammatory ^2^ effects; it can be applied as an anti-UV skin protector in the cosmetic industry.	[37,61,62]
Sandaracopimaradiene (tentatively identified) 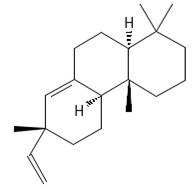	272.4681	Diterpene hydrocarbon with anti-inflammatory ^2^ and antiparasitic properties applied in traditional medicine, e.g., for respiratory and digestive illness treatment; as the antispasmodic agent, relief of smooth muscle spasms can be a therapeutic agent for, e.g., abdominal pain or intestinal cramps; inhibits the growth of *Staphylococcus aureus*, *Candida albicans*, and *Mycobacterium smegmatis* and acts as an antimalarial agent, inhibiting the growth of *Plasmodium falciparum*, the parasite responsible for malaria ^8^.	[37,63]
**triterpens**			
Squalene *** ** 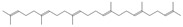 **	≈410.3912	The precursor of secondary metabolites; anticancer ^9^, anti-inflammatory ^2^, as well as cardioprotective and antioxidant agent ^1^.	[37,52]
**tocopherols**			
β-Tocopherol *** 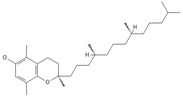	≈416.3654	Vitamin E has antioxidant ^1^, anticancer ^9^, or anti-heart disease properties by protecting cell membranes, their integrity, and their functionality; supports the immune system; improves skin health; and has anti-inflammatory ^2^ effects; it can be used as a dietary supplement ^3^.	[37,50,64]
**phytosterols**			
Campesterol ** 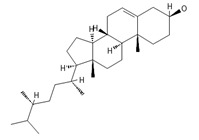 **	400.3705	Present in plants with antioxidant properties, it lowers low-density lipoprotein (LDL) cholesterol levels, reducing the risk of cardiovascular diseases and helping to prevent obesity ^4^, diabetes ^3^, and cancer ^9^. It also decreases the levels of metabolites such as β-carotene, lycopene, and vitamin E.	[37,65,66]
β-Sitosterol 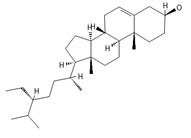	≈414.7180	A plant-derived sterol with antioxidant properties reduces LDL cholesterol levels, contributes to the improvement of heart health, and reduces the risk of cardiovascular diseases; it may reduce cancer ^9^ and digestive risks, e.g., of liver damage, by its anti-inflammatory and gastrointestinal protective properties.	[37,66,67]
Stigmasterol 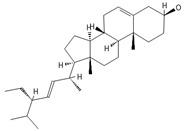	≈412.3705	A phytosterol with antioxidant ^1^ properties and cholesterol-lowering effects, it is suggested to have anticancer ^9^ properties by apoptosis induction, inflammatory bowel disease, stomach ulcers, and protecting the liver from toxin-induced damage.	[9,37,66]
**fatty acids**			
Hop-22(29)-ene *** (C30) 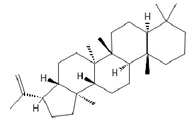	≈410.3913	Hop-22(29)-ene and its derivatives, such as 21αH-hop-22(29)-en-3-ol, are the agents with antioxidant ^1^ and anti-inflammatory ^2^ properties; biomedical applications in cancer ^9^ treatment and the production of dietary supplements; further clinical research is required.	[37,53]
Palmitic acid (C16) *** 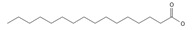	≈256.2402	Plays a key role in the palmitoylation of proteins and palmitoylated signal molecules.	[37,54]
**fatty acid esters**			
Methyl palmitate (C17) 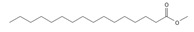	≈270.2558	Antioxidant ^1^, anti-inflammatory ^2^, anti-apoptotic, anti-fibrotic ^6^, and vasodilator ^7^ properties, as well as a cardioprotective agent.	[37,68,69]
Ethyl palmitate (C18) 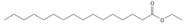	≈284.2715	Anti-inflammatory ^2^ and histoprotective effects.	[37,68]
Methyl stearate (C18) 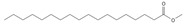	≈298.2871	Stearic acid ester is present in vegetable oils with skincare properties and is used in cosmetics and pharmaceuticals as well as a food stabilizer. It may cause allergic reactions.	[37,70]
Methyl behenate (C22) 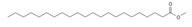	≈354.3497	Synthetic compound, substrate of behenyl behenate; applied in the cosmetic industry as a stabilizer and emulsifier.	[37]
**Lactones**			
Dihydroambrettolide 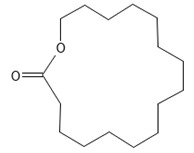	≈254.2290	High fragrance durability on the skin; pleasant-smelling compound; widely used in the perfume industry.	[37]
**Flavones**			
Schaftoside ** 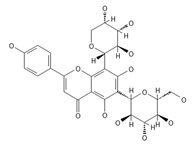 **	≈564.1479	Di-C-glycoside with antioxidative ^1^, anti-inflammatory ^2^, and anti-melanogenic activities, as well as antiepileptic properties.	[44,73]
Isoschaftoside, Apigenin 6-C-α-L-arabinoside 8-C-β-D-glucoside 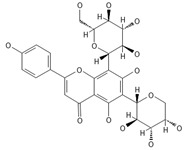	≈564.1479	Di-C-glycoside with neuroprotective and anti-inflammatory ^2^ properties.	[44,74]
Neoschaftoside 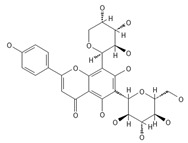	≈564.1479	Di-C-glycoside, a flavone derivative of apigenin with antimicrobial ^8^, anti-inflammatory, and anticancer ^9^ properties, can reduce the risk of cardiovascular disease.	[44,75]
Vicenin-2 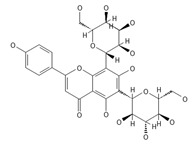	≈594.1584	Di-C-glycoside with anti-inflammatory ^2^ activities.	[44,76]
Chrysoeriol 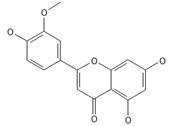	≈300.0634	In di-C-glycoside form, like neosisoschaftoside, present in, e.g., grains of purple barley, with high bioavailability after digestion, it is important for gastrointestinal health support.	[44,77]
Saponarin, luteolin 6-C-glycoside-7-O-glikozyd 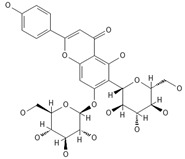	≈594.1580	Flavone-O-diglucoside, with antioxidant ^1^, anti-inflammatory ^2^, anti-allergic, and skin-protective properties, used in dietary supplements and cosmetics, may enhance mitochondrial metabolism.	[37,78,79]
**Biflavones**			
Amentoflavone ** 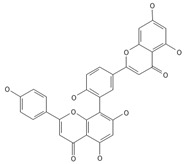 **	≈538.0900	Together with its derivatives like 7′-O-β-D-glucosylamentoflavone, exhibits antioxidant ^1^, anti-inflammatory ^2^, antiviral, antiseptic ^8^, and anticancer ^9^ properties.	[37,80]
Sequoiaflavone 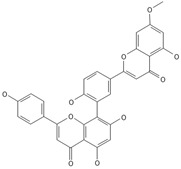	≈552.1056	Antioxidant ^1^, anti-inflammatory ^2^ antiviral, antiseptic ^8^, and anticancer ^9^ properties.	[37,80]
Bilobetin 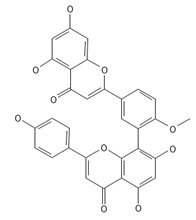	≈552.1056	Antioxidant ^1^, anti-inflammatory ^2^ antiviral, antiseptic ^8^, and anticancer ^9^ properties.	[37,80]
Podocarpusflavone 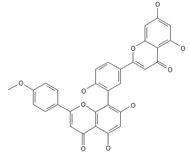	≈552.1056	Antioxidant ^1^, anti-inflammatory ^2^ antiviral, antiseptic ^8^, and anticancer ^9^ properties.	[37,80]
Ginkgetin 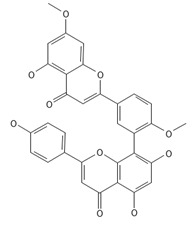	≈566.1213	The situation is similar for N-octadecane, although it is also not a unique metabolite for *P. affine*. It is an interesting cryoprotectant ^5^ and may be used in the cosmetics and pharmaceutical industries.	[37,80]
Isoginkgetin 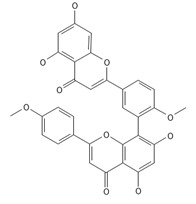	≈566.1213	Together with its glycoside derivative like 7′-O-β-D-glucosyl-isoginkgetin, exhibits antioxidant ^1^, anti-inflammatory ^2^ antiviral, antiseptic ^8^, and anticancer ^9^ properties.	[37,80]
5′-methoxybilobetine 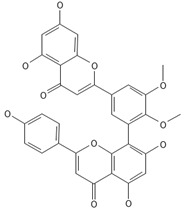	≈582.1162	Antioxidant ^1^, anti-inflammatory ^2^ antiviral, antiseptic ^8^, and anticancer ^9^ properties.	[37,80]
Sciadopitysins 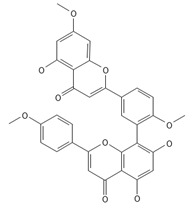	≈566.1110	Antioxidant ^1^, anti-inflammatory ^2^ antiviral, antiseptic ^8^, and anticancer ^9^ properties.	[37,80]
2,3-Dihydroisoginkgetin 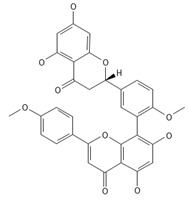	≈568.1370	Antioxidant ^1^, anti-inflammatory ^2^ antiviral, antiseptic ^8^, and anticancer ^9^ properties.	[37,80]
2,3-Dihydrosciadopitysin 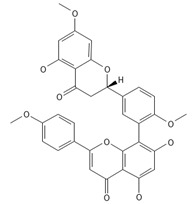	≈582.1553	Antioxidant ^1^, anti-inflammatory ^2^ antiviral, antiseptic ^8^, and anticancer ^9^ properties.	[37,80]

* The structure of the bioactive metabolites was prepared according to [81]. ** The molecular monoisotopic masses were verified and found to be consistent using the ChemSpider, PubChem, Chemical Book, LIPID MAPS^®^, NIST Chemistry WebBook, DrugBank, Aladdin Scientific, ChEBI, MetaCyc, and Phenol-Explorer databases. *** Metabolites are also indicated in *Plagiomnium affine* species.

**Table 3:** The antiproliferative effects of *P. affine* extracts [71]. Descriptions of extracts: initially all methanolic, then in n-hexane, chloroform, or 50% methanol. Values represent the percentage of cancer cell growth inhibition after 72 h exposure to the extracts; <25 indicates less than 25% inhibition, considered inactive. Higher values indicate stronger antiproliferative activity.

Extracts	HeLa 10 μg mL^−1^	HeLa 30 μg mL^−1^	A2780 10 μg mL^−1^	A2780 30 μg mL^−1^	T47D 10 μg mL^−1^	T47D 30 μg mL^−1^
A	<25	41.79	<25	<25	<25	42.41
B	55.53	<25	42.11	42.49	56.05	<25
C	42.04	50.67	<25	26.86	53.3	57.53
D	<25	<25	<25	<25	<25	<25

## Data Availability

The original contributions presented in this study are included in the article/Supplementary Material. Further inquiries can be directed to the corresponding author(s).

## Supplementary Materials

The following supporting information can be downloaded at https://www.mdpi.com/article/10.3390/ijms26199341/s1. References [89,90,91,92,93,94,95,96,97,98,99,100,101,102,103,104,105,106,107,108,109,110,111,112] are cited in Supplementary Materials.

## Abbreviations

The following abbreviations are used in this manuscript:

18S rDNA	Small subunit ribosomal RNA
26S rDNA	Large subunit ribosomal RNA
C	DNA content
CHCl_3_	Chloroform
cox1	Cytochrome c oxidase subunit 1
cpDNA	Chloroplast DNA
DMSO	Dimethylsulfoxide
GAPDH	Glyceraldehyde-3-phosphate dehydrogenase
ITS	Internal Transcribed Spacer
KN medium	Knop’s nutrient solution
matK	Maturase K gene
MS medium	Murashige and Skoog’s nutrient solution
mtDNA	Mitochondrial DNA
n	Numbers of chromosomes
nad5	NADH dehydrogenase subunit 5
PSII	Photosystem II
PHYP	Phytochrome genes
psbA-trnH	Spacer region between psbA and trnH genes
rDNA	Ribosomal DNA
rbcL	Ribulose-1,5-bisphosphate carboxylase large subunit
SL1344	One of *Salmonella enterica* serotype *Typhimurium*
trnL-F	tRNA-Leu and its spacer region
