# Supplementary material for: Molecular Insights into the Biomedical Applications of Plagiomnium affine (Blandow ex Funck) T. Kop.: A Promising Source of Bioactive Metabolites
Source: Int J Mol Sci. 2025 Sep 24;26(19):9341. doi: 10.3390/ijms26199341 (PMC12524726; doi:10.3390/ijms26199341)
Supplement: Supplementary file 1 [file ijms-26-09341-s001.zip › ijms-3733503-supplementary.pdf]

Supplementary Materials

Supplementary Box S1.

Antioxidant properties of agents. It is the ability of certain compounds, antioxidants e.g. polyphenols, to inhibit oxidation which can be responsible for formation of different types of free radicals, crucial in mitigating oxidative stress. Oxidative stress, for example, through DNA mutation, is linked to various chronic diseases and aging.
Antioxidants work by donating electrons to free radicals, stabilizing them by e.g. superoxide dismutase and glutathione peroxidase as well as other.

There are several types of free radicals, among others:

- Reactive Oxygen Species (ROS) – superoxide radical (O_2•$^-$) hydroxyl radical (•OH); hydrogen peroxide (H_2O_2); singlet oxygen ($^1O_2$).
- Reactive Nitrogen Species (RNS) – Nitric oxide radical (•NO), Peroxynitrite ($ONOO^-$).
- Reactive Sulfur Species (RSS) – Sulfanyl radical (•SH)
- Reactive Carbon Species (RCS) – Lipid peroxyl radical (LOO•); alkoxyl radical (RO•)

Literature

Antioxidants. Elsevier *eBooks* **2022**, 221–231. https://doi.org/10.1016/b978-0-323-91599-1.00007-9

Supplementary Box S2.

Anti-inflammatory properties of agents are related to the ability of a substance or treatment to reduce inflammation in the body. Inflammation is the body's natural response to injury, infection, or harmful stimuli, but chronic or excessive inflammation can lead to various diseases, including arthritis, cardiovascular diseases and autoimmune disorders.

Compounds with anti-inflammatory properties, such as nonsteroidal anti-inflammatory drugs (NSAIDs) or natural agents like curcumin (found in turmeric) or isoorientin, work by inhibiting the production of inflammatory mediators like cytokines, prostaglandins, and leukotrienes. These properties are essential for managing pain, swelling, and tissue damage in various medical conditions.

Literature

Inflammation: A multifaceted and omnipresent phenomenon. *Elsevier eBooks* **2023**, 19–30. https://doi.org/10.1016/b978-0-323-90960-0.00009-6

Özdemir, S. Inflammation: Complexity and significance of cellular and molecular responses. *J Acute Dis* **2024**, *13(1)*, 3–7. https://doi.org/10.4103/jad.jad_129_23

Supplementary Box S3.

Antidiabetic properties of agents are crucial in managing diabetes mellitus, a prevalent and life-threatening condition characterized by high blood glucose levels. These drugs or dietary supplements are designed to either increase insulin secretion, enhance insulin sensitivity or reduce glucose absorption, thereby controlling blood sugar levels and preventing complications such as neuropathy, nephropathy and cardiovascular diseases. Below are the key categories of antidiabetic agents and their mechanisms:

- Insulin and insulin analogues: important for Type 1 diabetes management (insulin-dependent, a chronic autoimmune disease in which the immune system attacks and destroys the beta cells in the pancreas). Insulin is injected or inhaled to compensate its production in the body.
- Oral hypoglycemic agents: these include biguanides, sulfonylureas, and meglitinides, which are primarily used for Type 2 diabetes (in which the pancreas produces insulin, but the cells become less responsive to it). They work by stimulating insulin secretion or improving insulin sensitivity.
- Dipeptidyl peptidase-4 (DPP-4) inhibitors: these agents enhance glucose-dependent insulin secretion from pancreatic β cells by preventing DPP-4-mediated degradation of endogenously released incretin hormones and decrease glucagon levels.
- Sodium-glucose cotransporter-2 (SGLT-2) inhibitors: they prevent glucose reabsorption in the kidneys thus, promotes its excretion through urine.
- Thiazolidinediones: these improve insulin sensitivity in muscle and fat tissues.
- Alpha-glucosidase inhibitors: they slow down carbohydrate absorption in the intestines, reducing postprandial blood glucose.
- Multi-target pharmacological actions: recent developments focus on drugs with multiple targets to enhance efficacy and reduce side effect.
- Adverse Effects and Drug Interactions: Despite their benefits, antidiabetic drugs can cause adverse reactions and interact with other medications, necessitating careful management.

Literature

Becker, R.; Trpin, B.; Brenner, M.; Hao, L.; Duncan, S.; Bégout, P. Anti-diabetic agents. *Elsevier eBooks* **2023** https://doi.org/10.1016/b978-0-12-824315-2.01134-9

Dowarah, J.; Singh, V. P. Anti-diabetic drugs: Recent approaches and advancements. *Bioorg Med Chem* **2020**, *28(5)*, 115263. https://doi.org/10.1016/J.BMC.2019.115263

Singh, V. P. An overview on anti-diabetic drugs and development. *Sci Tech J* **2016**, *4(2)*, 113–123. https://doi.org/10.22232/STJ.2016.04.02.05

Supplementary Box S4.

Anti-obesity agents are pharmacological or natural compounds designed to assist in weight management and combat obesity-related health issues. Recent advancements have highlighted both synthetic and natural agents, each with distinct mechanisms and efficacy profiles.

1. Synthetic GLP-1 receptor agonists: have shown significant weight loss and cardiovascular benefits. In a trial, semaglutide reduced body weight by 9.4% and decreased cardiovascular events in patients with preexisting conditions.
2. Synthetic dual agonists: tripeptide, targeting both GLP-1 and GIP receptors, is currently under investigation and shows promise as a potent anti-obesity agent.
3. Natural β-caryophyllene (BCP): this compound modulates gut microbiota, enhances energy expenditure, and inhibits lipid synthesis, making it a potential candidate for dietary supplements aimed at obesity management.

Literature

He, P.; Zang, L.; Zhu, S. New obesity treatment agent: β-Caryophyllene. *Curr Drug Ther* **2024**, 19. https://doi.org/10.2174/0115748855312851240823073921

Mikhail, N.; Wali, S. Semaglutide: The first anti-obesity agent shown to decrease cardiovascular events. *Ann Cardiovasc Dis* **2024**, *8(1)*, 1–4. https://doi.org/10.47739/2641-7731.cardiovasculardiseases.1036

Scheen, A.; Flines, J.; Paquot, N. Anti-obesity drugs: From previous disappointments to new hopes. *Rev Med Liège* **2023**, *78(3)*, 147–152.

Supplementary Box S5.

Cryoprotective agents designed to protect biological material during freezing and thawing, preventing ice crystal formation that can damage cells as well as to safeguard various nonbiological materials or surfaces from damage, degradation, or contamination.

- Medical applications are related used to preserve cells, tissues, sperm, eggs, and embryos for fertility treatments, stem cell therapy, and organ transplants.
- Industrial applications is used for food, pharmaceuticals, biotechnological and biological industry, which prevents ice crystal formation in frozen food, vaccines, enzymes, microorganisms and cultured cells during storage and transport.
- Industrial application is also related with protecting printed materials, plastic, and cables.

Literature

Ando, Y.; Nei, D.; Kono, S.; Nabetani, H. Current state and future issues of technology development concerned with freezing and thawing of foods. *J Jpn Soc Food Sci Technol* **2017**, *64(8)*, 391–428. https://doi.org/10.3136/NSKKK.64.391

Nishishita, N.; Muramatsu, M.; Kawamata, S. An effective freezing/thawing method for human pluripotent stem cells cultured in chemically defined and feeder-free conditions. **2015**

Pegg, D. E. Principles of cryopreservation. *Methods Cell Biol* **2007**, *82*, 39–57.

Supplementary Box S6.

Anti-fibrotic properties of agents, i.e. compounds designed to inhibit or reverse the pathological process of fibrosis (special type of degeneration), characterized by excessive extracellular matrix (ECM), deposition. (ECM is space between cell in human, which consists of collagens, elastin, proteoglycans, and glycosaminoglycans forming a network that helps maintain tissue integrity and supports their function). While the mentioned agents are crucial in managing various fibrotic diseases, including idiopathic pulmonary fibrosis and liver fibrosis.

Among the agents are polysaccharides, which modulate cell proliferation, inhibit inflammation, and regulate gut microbiota, demonstrating significant antifibrotic activity.

There are many anti-fibrotic agents, including herbal compounds, which inhibit the TGF-β signaling pathway.

Novel compounds like A8 target the Nur77 receptor, stabilizing it and preventing hepatic stellate cell activation.

Literature

Hong, W.; Xiao, T.; Lin, G.; Liu, C.; Li, H.; Li, Y.; Hu, H.; Wu, S.; Wang, S.; Liang, Z.; Lin, T.; Liu, J.; Chen, X. Structure-based design and synthesis of anti-fibrotic compounds derived from para-positioned 3,4,5-trisubstituted benzene. *Bioorg Chem* **2024**, *144*, 107113. https://doi.org/10.1016/j.bioorg.2024.107113

Ramadoss, R.; Sathish, S.; Sohn, H.; Madhavan, T. Potency of anti-fibrotic herbs on fibrogenesis: A theoretical evaluation. *Phytomedicine Plus* **2023**, *3(4)*, 100496. https://doi.org/10.1016/j.phyplu.2023.100496

Zhao, W.; Li, J.; Cai, J.; Gao, J.; Hu, Y. Research progress on the antifibrotic activity of traditional chinese medicine polysaccharides. Chem Biodivers **2024**, e202402012. https://doi.org/10.1002/cbdv.202402012

Supplementary Box S7.

Vasodilators properties of agents are related to widen blood vessels by relaxing the smooth muscles, leading to increased blood flow, lower blood pressure and improve circulation.

Among these agents are endogenous ones, nitric oxide (NO), prostacyclin (PGI2), Adenosine, and exogenous one nitrates, calcium channel blockers, ACE (angiotensin II, a hormone that constricts blood vessels inhibitors) and hydralazine.

Literature

Lepori, M.; Sartori, C.; Duplain, H.; Nicod, P.; Scherrer, U. Interaction between cholinergic and nitrergic vasodilation: a novel mechanism of blood pressure control. *Cardiovasc Res.* **2001**, *51(4)*, 767–772. https://doi.org/10.1016/S0008-6363(01)00325-X

Supplementary Box S8.

The antiseptic properties of agents are critical in preventing infections and managing wounds. Different compositions and mechanisms of action contribute to their effectiveness against a wide range of microorganisms, including bacteria, viruses, and fungi. These agents work by disrupting microbial cell membranes, denaturing proteins, or interfering with essential metabolic processes, ultimately leading to pathogen inactivation or destruction.

Antiseptics can be classified based on their active ingredients and target organisms. Alcohol-based antiseptics, such as ethanol and isopropanol, are highly effective against bacteria and enveloped viruses by denaturing proteins and dissolving lipids. Oxidizing agents, like hydrogen peroxide and iodine, exert their effect by generating reactive oxygen species that damage cellular components. Phenolic compounds, including triclosan and chlorhexidine, disrupt bacterial cell walls and inhibit enzyme activity, making them particularly effective in medical and dental applications. Quaternary ammonium compounds (QACs), such as benzalkonium chloride, function by disrupting microbial membranes, offering broad-spectrum antimicrobial activity.

Literature

Perera, K. C.; Ekanayaka, S. K.; Chandrasiri, N.; Jayatilleke, K.; Kottahachchi, J. In-vitro evaluation of bactericidal activity of antiseptics and disinfectants commonly used in healthcare settings. *Galle Medical Journal* **2021**, *26(1)*, 23. https://doi.org/10.4038/GMJ.V26I1.8079

Bäumler, W.; Eckl, D. B.; Holzmann, T.; Schneider-Brachert, W. Antimicrobial coatings for environmental surfaces in hospitals: a potential new pillar for prevention strategies in hygiene. *Critical Reviews in Microbiology* **2021**, 1–35. https://doi.org/10.1080/1040841X.2021.1991271

Hayek, S. N. Wound cleansing, topical antiseptics and wound. **2009** https://shadyhayek.com/wp-content/uploads/2012/02/Wound-cleansing-topical-antiseptics-and-wound-healing.pdf

Supplementary Box S9.

The anticancer properties of agents refer to their ability to prevent, inhibit the growth of, or treat cancer. These substances can act through various mechanisms, and their actions are diverse. In summary, anticancer properties may include:

1. Inhibition of cancer cell growth by block the processes of cell division in cancer cells arresting cells in specific phases, which impairs their further development and replication thereby reducing their ability to proliferate.
2. Induction of apoptosis (programmed cell death) by restore this process in the cancer cells, which is eliminated during carcinogenesis.
3. Inhibition of angiogenesis, the process of new blood vessel formation, which is crucial for tumor growth, as the new vessels supply nutrients and oxygen to the tumor. Anticancer substances can block angiogenesis, limiting the availability of resources for the growing tumor.
4. Modulation of the immune system in fighting cancer cells. By boosting the immune response, the body is better able to recognize and destroy cancerous cells.
5. Combatting oxidative stress, which is controlling the levels of reactive oxygen species (ROS) in cells. Excessive ROS can damage DNA and lead to cancer. Anticancer substances can act as antioxidants, reducing oxidative stress and preventing cellular damage.
6. Interaction in DNA of cancer cells inhibits DNA replication, which can lead to the induction of apoptosis and death of cancer cells.

Metabolites with anticancer effects include chemotherapeutic drugs such as cisplatin, as well as plant-derived compounds like curcumin (from turmeric) and resveratrol (from grapes). Vitamins, minerals, and plant-based compounds with anti-inflammatory and antioxidant properties are also often used to support cancer treatment.

Literature

Hanahan, D.; Weinberg, R. A. Hallmarks of cancer: The next generation. *Cell* **2011**, *144(5)*, 646-674. https://doi.org/10.1016/j.cell.2011.02.013

Vaux, D. L.; Korsmeyer, S. J. Cell death in development. *Cell* **1999**, *96(2)*, 245-254. https://doi.org/10.1016/S0092-8674(00)80556-9

Carmeliet, P.; Jain, R. K. Angiogenesis in cancer and other diseases. *Nature* **2000**, *407(6801)*, 249-257. https://doi.org/10.1038/35025220

Sies, H.; Jones, D. P. Oxidative stress. *Annual Review of Biochemistry* **2020**, *89*, 1-20. https://doi.org/10.1146/annurev-biochem-010419-060833

Nitiss, J. L. Targeting DNA topoisomerase II in cancer chemotherapy. *Nature Reviews Cancer* **2009**, *9(5)*, 338-350. https://doi.org/10.1038/nrc2628
